# Fully Automated EUCAST Rapid Antimicrobial Susceptibility Testing (RAST) from Positive Blood Cultures: Diagnostic Accuracy and Implementation

**DOI:** 10.1128/jcm.00898-22

**Published:** 2022-09-29

**Authors:** Abdessalam Cherkaoui, Didier Schorderet, Nouria Azam, Luigi Crudeli, José Fernandez, Gesuele Renzi, Adrien Fischer, Jacques Schrenzel

**Affiliations:** a Bacteriology Laboratory, Division of Laboratory Medicine, Department of Diagnostics, Geneva University Hospitals, Geneva, Switzerland; b Faculty of Medicine, Geneva, Switzerland; c Genomic Research Laboratory, Division of Infectious Diseases, Department of Medicine, Geneva University Hospitals and Faculty of Medicine, Geneva, Switzerland; NorthShore University HealthSystem

**Keywords:** full automation, RAST, AST, WASPLab, Copan Radian, blood cultures, EUCAST RAST

## Abstract

The objective of this study was to evaluate the accuracy and robustness of a fully automated EUCAST RAST (rapid antimicrobial susceptibility test) directly from positive blood culture and to appreciate its implementation constraints. This study was conducted in two phases: (i) spiked blood culture bottles (BCs) using 779 non-duplicate clinical isolates and (ii) a prospective clinical trial including 534 positive BCs sequentially processed in routine at the Bacteriology Laboratory of Geneva University Hospitals. The RAST results were assessed against EUCAST standardized disk diffusion testing results. Our first finding was that the results of the spiked BCs precisely predicted the clinical trial results. The overall categorical agreements for all species analyzed were greater than 95% at the different time points. RAST for Pseudomonas aeruginosa, however, raised several challenges. The categorical agreement for imipenem was lower than 95% at 6 h and was not improved with longer incubation times. Additionally, piperacillin-tazobactam, ceftazidime, and cefepime cannot be released at 6 h due to suboptimal performances, but the categorical agreement substantially improved at 8 h. Our results establish that the performance of fully automated EUCAST RAST directly from positive blood culture bottles is consistently robust, even for the detection of extended-spectrum β-lactamase (ESBL), carbapenemase-producing bacteria, and methicillin-resistant Staphylococcus aureus (MRSA). The automation markedly enhanced the percentage of readable inhibition zones and reduced the percentage of isolates categorized in the area of technical uncertainty (ATU). In summary, a fully automated EUCAST RAST can substantially improve laboratory workflow by reducing hands-on time and removing the strong constraints linked to manual read-outs at precisely defined times.

## INTRODUCTION

Over the last few years, many RAST methods performed directly on positive blood culture bottles were developed with the overwhelming objective of reducing turn-around times (TAT). Different reports have highlighted the strengths and weaknesses of each method, with special attention paid to a panel of testable microorganisms and accuracy for detecting various antimicrobial resistance mechanisms (1–6). The value of the RAST has never been more important than it is now, with the large spread of multidrug-resistant (MDR) and extremely drug-resistant (XDR) bacteria among humans, animals, and environmental reservoirs. Consequently, invasive infections caused by these bacteria are increasing substantially over time. Furthermore, it has been well documented that the outcomes of patients suffering from bloodstream infection and septic shock are noticeably influenced by the rapid administration of effective antimicrobial treatment (7–10).

The introduction of matrix-assisted laser desorption ionization–time of flight mass spectrometry (MALDI-TOF/MS) in clinical microbiology has provided a major step forward, enabling faster microorganism identification. Regarding antimicrobial susceptibility testing (AST), however, certain obstacles must be overcome before routine implementation of faster methods. Full laboratory automation has permitted the disk diffusion method to return to center stage and strengthen its position among other AST methods currently used in clinical microbiology laboratories. Exquisite flexibility, cost-effectiveness, and accuracy for detecting new resistance mechanisms remain the major advantages of the disk diffusion method (11). Over the last 3 years, EUCAST has developed a standardized rapid method based on disk diffusion to enable susceptibility reports within 4 to 8 h and 16 to 18 h directly from positive blood culture bottles (BCs). Although this method provides faster and more accurate results, its manual setup and the imperative requirement to read the inhibition zone diameters at strictly defined time points are tremendously labor-intensive and have thus hampered its large-scale use in clinical microbiology laboratories, not to mention routine usage (12).

The main objective of this study was to evaluate the accuracy and robustness of the fully automated rapid antimicrobial disk diffusion susceptibility testing (RAST, EUCAST) directly from positive blood culture bottles and to appreciate its implementation constraints. This study was undertaken in two phases. Phase 1 consisted of spiked blood culture bottles including various resistant phenotypes, including MDR strains, according to the procedure described by Jonasson et al. for the development of the EUCAST RAST method. Phase 2 was a prospective clinical study which sequentially included positive BCs processed in our routine laboratory.

## MATERIALS AND METHODS

### Clinical bacterial isolates.

The bacterial isolates included in the spiking phase consisted of 779 non-duplicate isolates (207 Escherichia coli, 118 Klebsiella pneumoniae, 129 Pseudomonas aeruginosa, 63 Acinetobacter baumannii, 148 Staphylococcus aureus, 66 Enterococcus faecalis, and 48 Enterococcus faecium) identified from non-consecutive clinical samples referred to our laboratory. Carbapenemase-producing Enterobacterales (CPE), multidrug-resistant (MDR) A. baumannii and P. aeruginosa, vancomycin-resistant E. faecalis and E. faecium (VRE), and S. aureus isolates with inducible clindamycin resistance were previously identified and stored at −80°C in skim milk with 15% glycerol. All stored isolates were passaged twice before testing. Table 1 shows the different phenotypes of the bacterial isolates analyzed in the spiking phase. Identification of strains was carried out by matrix-assisted laser desorption ionization–time of flight mass spectrometry (MALDI-TOF/MS) (MBT Compass 4.1, library version 11.0 [11,410 spectra], Bruker Daltonics, Bremen, Germany) according to the manufacturer’s instructions.

**TABLE 1:** Phenotypes of the bacterial isolates analyzed in the spiked BCs phase^*a*^

Phenotype (*n*)	Species						
	*E. coli*	*K. pneumoniae*	*P. aeruginosa*	*A. baumannii*	*S. aureus*	*E. faecalis*	*E. faecium*
ESBL	51	50					
OXA-23-like producers				27			
OXA-48-like producers	3	7					
OXA-58-like producers	1			2			
OXA-181-like producers	10	1					
KPC-like producers	1	12					
NDM-like producers	2	13		7			
VIM-like producers			2				
MRSA					17		
Inducible clindamycin resistance					47		
VanA producers^*b*^						6	14
VanB producers^*c*^							9
High-level gentamicin resistance						16	20
Total^*d*^	207	118	129	63	148	66	48

a BC, blood culture; ESBL, extended-spectrum β-lactamase; MRSA, methicillin-resistant *Staphylococcus aureus*.

b For the isolates with the VanA phenotype, vancomycin MICs ranged between 32 and 256 mg/L.

c For the isolates with the VanB phenotype, vancomycin MICs ranged between 6 and 256 mg/L.

d Some isolates expressed more than one phenotype and are only counted once.

The extended-spectrum β-lactamase (ESBL) profile was confirmed by a double-disk synergy test (DDST20). For this confirmation test, an amoxicillin-clavulanate disk was automatically placed at 20 mm, center to center, of a cefepime disk on Mueller-Hinton E (MHE) agar, according to a previously reported method (13). The Eazyplex SuperBug CRE system (Amplex Biosystems GmbH, Giessen, Germany) was used to identify the genes encoding carbapenemases. The presence of methicillin-resistant Staphylococcus aureus (MRSA) was confirmed by a previously published quantitative PCR (qPCR) assay targeting *femA* and *mecA* (14). For the vancomycin-resistant enterococci, the *vanA* and *vanB* genes were identified by qPCR assays. EUCAST standardized disk diffusion testing was performed on all the isolates included in the present study using the fully automated solution which was previously validated and has been implemented in our routine since February 2021 (11). The inhibition zone diameters were interpreted according to the EUCAST Breakpoint Tables version 11.0.

### Spiking blood culture bottles.

We rigorously followed the procedure described by Jonasson et al. as used for the development of the EUCAST RAST method (15). To mimic real routine conditions, we used patients’ negative BC bottles which had been previously analyzed in our lab. The BC bottles (BD BACTEC Plus Aerobic medium, BD BACTEC Lytic Anaerobic medium, or BD BACTEC Peds Plus) reported negative on day 5 were inoculated with a bacterial suspension of 100 to 200 CFU of an overnight culture from a Columbia blood agar plate. Inoculated bottles were immediately placed in a Bactec FX instrument (Becton, Dickinson and Company). BC bottles turned positive after 3 to 16 h of incubation in the instrument (average time: 10.5 h). BC bottles were removed from the Bactec FX 15 min to 13 h after growth was detected (average time: 4 h) according to the working hours of the lab. Fully automated disc diffusion was performed immediately according to the EUCAST RAST method and the manufacturer’s instructions (Copan) (Fig. S1 in the supplemental material). We used 90-mm circular plates, MHE agar, (bioMérieux, Geneva, Switzerland), and i2a antibiotic disks (i2a, Montpellier, France). Several high-resolution digital images were acquired under different light and exposure conditions at defined time points (4, 6, and 8 h).

Inhibition zone diameters were automatically derived by the WASPLab for confluent growth with distinctly delineated margins. All the inhibition zone diameters were validated on the WASPLab screen by microbiologists and experienced technologists. Therefore, there was no automatic release of the results. Inhibition zone diameters were modified manually whenever appropriate or necessary, which concerned about 5% of all tested disks.

### RAST results assessment.

For each reading time, the Copan expert system interpreted the inhibition zone diameters validated on the WASPLab screen by microbiologists and experienced technologists as S, R, or area of technical uncertainty (ATU) according to the RAST EUCAST Breakpoint Tables version 3.0. All RAST results, where an interpretation of S or R was defined, were rigorously compared to the results of EUCAST standardized disk diffusion (DD) testing. The number of very major errors (VMEs; RAST = S, standardized DD = R), major errors (MEs; RAST = R and standardized DD = S), and minor errors (mEs; RAST = S or R and standardized DD = intermediate, I) were established. The proportion of the tests validated as ATU and the tests with no readable inhibition zones were determined for the different time points (4, 6, and 8 h).

### Bacterial cells density.

For the spiked BCs, we deemed it relevant to assess the bacterial cell density when growth was detected by the instrument. Therefore, the final bacterial cell densities for 10 separate spiked BCs were assessed by viable-cell counts on Columbia agar after overnight incubation.

The bacterial cell density of the positive BCs was determined between 30 min to 2 h after growth was detected by the Bactec FX instrument. Bacterial cell densities ranged between 1.3 × 10^8^ and 5.6 × 10^8^ CFU/mL. This range included all the species analyzed (Gram-positive and Gram-negative bacteria).

### Clinical trial.

The clinical trial prospectively included all patients’ positive BCs received at our lab between December 2021 and May 2022, except for polymicrobial BCs and positive BCs with other bacterial species not included in the RAST EUCAST Breakpoint Tables version 3.0. These positive BCs were excluded post-RAST setup. Based on these specifications, all positive BCs from each patient were included with the aim of evaluating the repeatability and reproducibility of the RAST method. In total, 534 positive BCs were analyzed in the clinical trial. The RAST was carried out using the positive blood culture broths when they initially signaled positive. We defined a panel of antibiotics covering all bacterial species analyzed in this study. Thereby, we performed the RAST without waiting for the microscopy results. The non-pertinent antibiotics according to the identified bacterial species were excluded from the analysis and reporting during the reading phase. The identification of organisms isolated from the positive blood culture was carried out by MALDI-TOF/MS using a previously defined procedure (16). This identification was always available before RAST interpretation. Table 2 depicts the number of positive BCs broken down by species and phenotype of the isolates tested.

**TABLE 2:** Independent positive BC broken down by species and phenotype^*a*^

Phenotype (*n*)	Species						
	*E. coli*	*K. pneumoniae*	*P. aeruginosa*	*A. baumannii*	*S. aureus*	*E. faecalis*	*E. faecium*
Isolates	198	80	12	2	122	46	40
ESBL	18	3					
OXA-48-like producers		1					
MRSA					3		
Inducible clindamycin resistance					2		
VanB producers^*b*^							1
High-level gentamicin resistance						4	3
Total^*c*^	216	83	12	2	127	50	44

a BC, blood culture; ESBL, extended-spectrum β-lactamase; MRSA, methicillin-resistant *Staphylococcus aureus*.

b For the isolate with the VanB phenotype, the vancomycin MIC was 16 mg/L.

c Some isolates express more than one phenotype and are only counted once.

### Quality control.

Quality control (QC) of the RAST method was performed by spiking patients’ negative BC bottles with E. coli ATCC 25299 and S. aureus ATCC 29213. We followed the same methodology as described above. QC was repeated, throughout the experiments, 12 and 9 times for E. coli ATCC 25299 and S. aureus ATCC 29213, respectively.

### Detection of resistance mechanisms by RAST.

**(i) ESBL and carbapenemases.** Screening for ESBL and carbapenemases using the RAST method was carried out according to EUCAST RAST screening cutoff values at 4, 6 and 8 h (version 1).

We decided to systematically include a supplement MHE plate to the RAST AST panel defined for E. coli and K. pneumoniae. This was to assess the performance of the double-disk synergy test (DDST20) for the confirmation of ESBL producers at 4, 6, and 8 h of incubation using the RAST method. As for the standardized disk diffusion testing, an amoxicillin-clavulanate disk was automatically placed at 20 mm, center to center, of a cefepime disk on MHE agar. This test was considered positive when the inhibition zone around the cefepime disk was enhanced.

**(ii) Inducible clindamycin resistance.** To assess the performances of inducible clindamycin resistance test with the RAST method at 4, 6, and 8 h of incubation, we followed two protocols: a (i) clindamycin 2-μg disk and an erythromycin 15-μg disk placed 9 mm apart (edge to edge), and a (ii) clindamycin 2-μg disk and an erythromycin 15-μg disk placed 12 mm apart (edge to edge). These protocols were applied on 49 non-duplicate erythromycin-resistant S. aureus isolates, including 10 MRSA. For all of these 49 isolates, the D phenomenon was positive with standardized disk diffusion testing. Additional erythromycin and clindamycin disks were tested separately in the RAST AST panel for S. aureus.

## RESULTS

### Readable inhibition zones.

The percentages of inhibition zones with no growth, no confluent growth, or no distinctly delineated growth at the different time points were assessed for each species during the two study phases (spiked BCs and clinical trial), as shown in Tables 3 to 14. After 4 h of incubation, E. coli, K. pneumoniae, and A. baumannii grew well. The proportion of readable zones at 4 h for all the antibiotics tested during the spiked phase was 96.6% (1,600/1,656) for E. coli, 100% for K. pneumoniae (944/944), and 96.8% (427/441) for A. baumannii. This proportion reached 100% at 6 h. For P. aeruginosa, the proportion of readable zones at 6 h was 96.9% (1,125/1,161) and attained 100% at 8 h. As expected, Gram-positive bacteria required more time to provide readable inhibition zones. For S. aureus, this rate was 87.3% (517/592) at 4 h and 100% at 6 h. For E. faecalis, this rate was 93.9% (310/330) at 4 h and 100% at 6 h. The same figures were observed during the clinical trial.

**TABLE 3:** *Escherichia coli* spiked BC results (*n* = 207 isolates)^*a*^

*E. coli* spiked BC parameters	Piperacillin-tazobactam			Ceftazidime			Imipenem			Meropenem			Ciprofloxacin			Gentamicin			Amikacin			Co-trimoxazole		
	4 h	6 h	8 h	4 h	6 h	8 h	4 h	6 h	8 h	4 h	6 h	8 h	4 h	6 h	8 h	4 h	6 h	8 h	4 h	6 h	8 h	4 h	6 h	8 h
Isolates by category (*n*)																								
Resistant	30	36	34	8	21	32	6	11	9	7	9	9	56	63	62	24	26	26	33	14	12	69	74	74
ATU	81	54	51	23	13	7	3	6	7	8	5	4	20	23	16	5	3	2	128	83	54	2	1	2
Susceptible	89	117	122	169	173	168	191	190	191	185	193	194	124	121	129	171	178	179	39	110	141	129	132	131
Inhibition zone not readable	7	0	0	7	0	0	7	0	0	7	0	0	7	0	0	7	0	0	7	0	0	7	0	0
Isolates by category (%)																								
Resistant	15.0	17.4	16.4	4.0	10.1	15.5	3.0	5.3	4.3	3.5	4.3	4.3	28.0	30.4	30.0	12.0	12.6	12.6	16.5	6.8	5.8	34.5	35.7	35.7
ATU	40.5	26.1	24.6	11.5	6.3	3.4	1.5	2.9	3.4	4.0	2.4	1.9	10.0	11.1	7.7	2.5	1.4	1.0	64.0	40.1	26.1	1.0	0.5	1.0
Susceptible	44.5	56.5	58.9	84.5	83.6	81.2	95.5	91.8	92.3	92.5	93.2	93.7	62.0	58.5	62.3	85.5	86.0	86.5	19.5	53.1	68.1	64.5	63.8	63.3
Inhibition zone not readable	3.4	0.0	0.0	3.4	0.0	0.0	3.4	0.0	0.0	3.4	0.0	0.0	3.4	0.0	0.0	3.4	0.0	0.0	3.4	0.0	0.0	3.4	0.0	0.0
Categorical agreement (%)^*b*^																								
ATU included	98.5	98.1	98.6	98.0	98.1	98.6	100.0	99.5	99.5	99.5	99.5	99.5	99.5	99.5	99.5	99.5	98.1	98.1	94.0	98.1	97.6	98.0	97.6	97.6
ATU not included	97.5	97.4	98.1	97.7	97.9	98.5	100.0	99.5	99.5	99.5	99.5	99.5	99.4	99.5	99.5	99.5	98.0	9 8.0	83.3	96.8	96.7	98.0	97.6	97.6
Discordant results (*n*)																								
Minor error				1	1	1																		
Major error	1	2	1					1	1	1	1	1		1	1		1	1	10	1	1	4	5	5
Very major error	2	2	2	3	3	2							1			1	3	3	2	3	4			
Discordant results (%)																								
Minor error	0.0	0.0	0.0	0.5	0.5	0.5	0.0	0.0	0.0	0.0	0.0	0.0	0.0	0.0	0.0	0.0	0.0	0.0	0.0	0.0	0.0	0.0	0.0	0.0
Major error	0.5	1.0	0.5	0.0	0.0	0.0	0.0	0.5	0.5	0.5	0.5	0.5	0.0	0.5	0.5	0.0	0.5	0.5	4.8	0.5	0.5	1.9	2.4	2.4
Very major error	1.0	1.0	1.0	1.4	1.4	1.0	0.0	0.0	0.0	0.0	0.0	0.0	0.5	0.0	0.0	0.5	1.4	1.4	1.0	1.4	1.9	0.0	0.0	0.0

a BC, blood culture; ATU, area of technical uncertainty. Very major error, RAST = S and reference AST = R; major error, RAST = R and reference AST = S; minor error, RAST = S or R and reference AST = I.

b Categorical agreement for RAST at 4, 6 and 8 h of incubation compared to EUCAST standardized disk diffusion testing.

**TABLE 4:** *Escherichia coli* clinical trial results (*n* = 216 independent positive BCs from 87 patients)^*a*^

*E. coli* clinical trial parameters	Piperacillin-tazobactam			Ceftazidime			Imipenem			Meropenem			Ciprofloxacin			Gentamicin			Amikacin			Co-trimoxazole		
	4 h	6 h	8 h	4 h	6 h	8 h	4 h	6 h	8 h	4 h	6 h	8 h	4 h	6 h	8 h	4 h	6 h	8 h	4 h	6 h	8 h	4 h	6 h	8 h
Isolates by category (*n*)																								
Resistant	16	27	28	18	29	31	0	0	0	0	0	0	54	57	57	26	26	27	20	10	10	93	99	100
ATU	117	76	65	26	5	4	0	0	0	3	1	1	9	24	18	12	2	1	159	105	60	5	1	0
Susceptible	75	113	123	164	182	181	208	216	216	205	215	215	145	135	141	170	188	188	29	101	146	110	116	116
Inhibition zone not readable	8	0	0	8	0	0	8	0	0	8	0	0	8	0	0	8	0	0	8	0	0	8	0	0
Isolates by category (%)																								
Resistant	7.7	12.5	13.0	8.7	13.4	14.4	0.0	0.0	0.0	0.0	0.0	0.0	26.0	26.4	26.4	12.5	12.0	12.5	9.6	4.6	4.6	44.7	45.8	46.3
ATU	56.3	35.2	30.1	12.5	2.3	1.9	0.0	0.0	0.0	1.4	0.5	0.5	4.3	11.1	8.3	5.8	0.9	0.5	76.4	48.6	27.8	2.4	0.5	0.0
Susceptible	36.1	52.3	56.9	78.8	84.3	83.8	100.0	100.0	100.0	98.6	99.5	99.5	69.7	62.5	65.3	81.7	87.0	87.0	13.9	46.8	67.6	52.9	53.7	53.7
Inhibition zone not readable	3.7	0.0	0.0	3.7	0.0	0.0	3.7	0.0	0.0	3.7	0.0	0.0	3.7	0.0	0.0	3.7	0.0	0.0	3.7	0.0	0.0	3.7	0.0	0.0
Categorical agreement (%)^*b*^																								
ATU included	99.5	99.5	99.1	100.0	100.0	100.0	100.0	100.0	100.0	100.0	100.0	100.0	99.0	99.5	99.5	99.5	99.5	99.5	98.1	98.1	98.1	99.5	100.0	100.0
ATU not included	98.9	99.3	98.7	100.0	100.0	100.0	100.0	100.0	100.0	100.0	100.0	100.0	99.0	99.5	99.5	99.5	99.5	99.5	91.8	96.4	97.4	99.5	100.0	100.0
Discordant results (*n*)																								
Major error													1			1	1	1	4					
Very major error	1	1	2										1	1	1					4	4	1		
Discordant results (%)																								
Major error	0.0	0.0	0.0	0.0	0.0	0.0	0.0	0.0	0.0	0.0	0.0	0.0	0.5	0.0	0.0	0.5	0.5	0.5	1.9	0.0	0.0	0.0	0.0	0.0
Very major error	0.5	0.5	0.9	0.0	0.0	0.0	0.0	0.0	0.0	0.0	0.0	0.0	0.5	0.5	0.5	0.0	0.0	0.0	0.0	1.9	1.9	0.5	0.0	0.0

a BC, blood culture; ATU, area of technical uncertainty. Very major error, RAST = S and reference AST = R; major error, RAST = R and reference AST = S; minor error, RAST = S or R and reference AST = I.

b Categorical agreement for RAST at 4, 6 and 8 h of incubation compared to EUCAST standardized disk diffusion testing.

### Area of technical uncertainty.

According to the EUCAST RAST breakpoints (version 3.0), the percentage of ATU by antibiotic and species are shown in Tables 3 to 14 for the different time points and the two study phases. The percentage of ATU decreased with the incubation time in both study phases. For Gram-negative bacteria, the most affected molecules were piperacillin-tazobactam, amikacin, ciprofloxacin, and cotrimoxazole. For S. aureus, gentamicin and norfloxacin were concerned.

### Results according to the EUCAST RAST breakpoints (version 3.0).

**E. coli**. For the spiked BCs, incorrect categorization was very low despite the fact that we included a large number of isolates with diverse resistance phenotypes. Several MEs were observed for amikacin, but most disappeared at 6 h. This coincided with unexpected high rates of ATU for this antibiotic at 4 h. The percentage of ATU for piperacillin-tazobactam was also high (40.5% at 4 h), but it was almost halved at 6 and 8 h. Few errors were observed for this antibiotic and the categorical agreement exceeded 97% at 4 h. Categorical agreement for RAST at 4 h of incubation compared with the EUCAST standardized disk diffusion test results was >95% for all the antibiotics tested, with the exception of amikacin (Table 3). The overall categorical agreements were 98.4%, 98.5%, and 98.6% at 4, 6, and 8 h, respectively.

The same observations were made in the clinical trial, but with even lower rates of incorrect categorization. This can be partly explained by the selection of resistant phenotypes during the first phase. There were no carbapenem-resistant isolates and only a few ESBL in the second phase. The overall categorical agreements for all antibiotics tested during the clinical trial were 99.5%, 99.6% and 99.6% at 4, 6, and 8 h, respectively (Table 4).

**K. pneumoniae**. Categorical agreement for RAST at 4 h of incubation compared with the EUCAST standardized disk diffusion test results was >95% for all antibiotics tested with spiked BCs (Table 5). The overall categorical agreements with spiked BCs were 99.0%, 98.7%, and 99.0% at 4, 6, and 8 h, respectively. The few VMEs and MEs were not related to any specific drug. Importantly, 100% of the inhibition zones were readable at 4 h. As noticed for E. coli, a high rate of ATU was observed for amikacin at 4 h, but this rate declined over time. The clinical trial results supported the observations made with the spiked BCs, with the exception of piperacillin-tazobactam. Some VMEs and MEs were identified for this antibiotic at different time points during the clinical trial. Therefore, categorical agreement was <95% for the three time points. The overall categorical agreements for all antibiotics tested during the clinical trial were 98.8%, 99.1%, and 99.1% at 4, 6, and 8 h, respectively (Table 6).

**TABLE 5:** *Klebsiella pneumoniae* spiked BC results (*n* = 118 isolates)^*a*^

*K. pneumoniae* spiked BC parameters	Piperacillin-tazobactam			Ceftazidime			Imipenem			Meropenem			Ciprofloxacin			Gentamicin			Amikacin			Cotrimoxazole		
	4 h	6 h	8 h	4 h	6 h	8 h	4 h	6 h	8 h	4 h	6 h	8 h	4 h	6 h	8 h	4 h	6 h	8 h	4 h	6 h	8 h	4 h	6 h	8 h
Isolates by category (*n*)																								
Resistant	45	51	52	46	50	49	27	27	30	24	30	31	41	36	44	29	30	27	23	13	22	43	43	46
ATU	23	18	15	4	2	3	3	4	1	5	4	3	18	16	9	5	3	6	50	13	27	4	4	1
Susceptible	50	49	51	68	66	66	88	87	87	89	84	84	59	66	65	84	85	85	45	92	69	71	71	71
Inhibition zone not readable	0	0	0	0	0	0	0	0	0	0	0	0	0	0	0	0	0	0	0	0	0	0	0	0
Isolates by category (%)																								
Resistant	38.1	43.2	44.1	39.0	42.4	41.5	22.9	22.9	25.4	20.3	25.4	26.3	34.7	30.5	37.3	24.6	25.4	22.9	19.5	11.0	18.6	36.4	36.4	39.0
ATU	19.5	15.3	12.7	3.4	1.7	2.5	2.5	3.4	0.8	4.2	3.4	2.5	15.3	13.6	7.6	4.2	2.5	5.1	42.4	11.0	22.9	3.4	3.4	0.8
Susceptible	42.4	41.5	43.2	57.6	55.9	55.9	74.6	73.7	73.7	75.4	71.2	71.2	50.0	55.9	55.1	71.2	72.0	72.0	38.1	78.0	58.5	60.2	60.2	60.2
Inhibition zone not readable	0.0	0.0	0.0	0.0	0.0	0.0	0.0	0.0	0.0	0.0	0.0	0.0	0.0	0.0	0.0	0.0	0.0	0.0	0.0	0.0	0.0	0.0	0.0	0.0
Categorical agreement (%)^*b*^																								
ATU included	96.6	96.6	96.6	100.0	100.0	100.0	100.0	100.0	99.2	99.2	99.2	98.3	100.0	100.0	100.0	99.2	100.0	99.2	99.2	95.8	100.0	98.3	98.3	98.3
ATU not included	95.8	96.0	96.1	100.0	100.0	100.0	100.0	100.0	99.1	99.1	99.1	98.3	100.0	100.0	100.0	99.1	100.0	99.1	98.5	95.2	100.0	98.2	98.2	98.3
Discordant results (*n*)																								
Major error									1	1	1	2							1			1	1	1
Very major error	4	4	4													1		1		5		1	1	1
Discordant results (%)																								
Major error	0.0	0.0	0.0	0.0	0.0	0.0	0.0	0.0	0.8	0.8	0.8	1.7	0.0	0.0	0.0	0.0	0.0	0.0	0.8	0.0	0.0	0.8	0.8	0.8
Very major error	3.4	3.4	3.4	0.0	0.0	0.0	0.0	0.0	0.0	0.0	0.0	0.0	0.0	0.0	0.0	0.8	0.0	0.8	0.0	4.2	0.0	0.8	0.8	0.8

a BC, blood culture; ATU, area of technical uncertainty. Very major error, RAST = S and reference AST = R; major error, RAST = R and reference AST = S; minor error, RAST = S or R and reference AST = I.

b Categorical agreement for RAST at 4, 6 and 8 h of incubation compared to EUCAST standardized disk diffusion testing.

**TABLE 6:** *Klebsiella pneumoniae* clinical trial results (*n* = 83 independent positive BCs from 29 patients)^*a*^

*K. pneumoniae* clinical trial parameters	Piperacillin-tazobactam			Ceftazidime			Imipenem			Meropenem			Ciprofloxacin			Gentamicin			Amikacin			Cotrimoxazole		
	4 h	6 h	8 h	4 h	6 h	8 h	4 h	6 h	8 h	4 h	6 h	8 h	4 h	6 h	8 h	4 h	6 h	8 h	4 h	6 h	8 h	4 h	6 h	8 h
Isolates by category (*n*)																								
Resistant	9	13	12	6	5	6	1	1	1	1	1	1	5	4	6	4	4	4	2	1	0	13	14	14
ATU	22	21	23	10	8	7	2	1	1	0	0	1	17	14	13	8	5	2	40	3	20	0	0	0
Susceptible	51	49	48	66	70	70	79	81	81	81	82	81	60	65	64	70	74	77	40	79	63	69	69	69
Inhibition zone not readable	1	0	0	1	0	0	1	0	0	1	0	0	1	0	0	1	0	0	1	0	0	1	0	0
Isolates by category (%)																								
Resistant	11.0	15.7	14.5	7.3	6.0	7.2	1.2	1.2	1.2	1.2	1.2	1.2	6.1	4.8	7.2	4.9	4.8	4.8	2.4	1.2	0.0	15.9	16.9	16.9
ATU	26.8	25.3	27.7	12.2	9.6	8.4	2.4	1.2	1.2	0.0	0.0	1.2	20.7	16.9	15.7	9.8	6.0	2.4	48.8	3.6	24.1	0.0	0.0	0.0
Susceptible	62.2	59.0	57.8	80.5	84.3	84.3	96.3	97.6	97.6	98.8	98.8	97.6	73.2	78.3	77.1	85.4	89.2	92.8	48.8	95.2	75.9	84.1	83.1	83.1
Inhibition zone not readable	1.2	0.0	0.0	1.2	0.0	0.0	1.2	0.0	0.0	1.2	0.0	0.0	1.2	0.0	0.0	1.2	0.0	0.0	1.2	0.0	0.0	1.2	0.0	0.0
Categorical agreement (%)^*b*^																								
ATU included	92.7	94.0	94.0	100.0	100.0	100.0	100.0	100.0	100.0	100.0	100.0	100.0	98.8	98.8	98.8	100.0	100.0	100.0	98.8	100.0	100.0	100.0	100.0	100.0
ATU not included	90.0	91.9	91.7	100.0	100.0	100.0	100.0	100.0	100.0	100.0	100.0	100.0	98.5	98.6	98.6	100.0	100.0	100.0	97.6	100.0	100.0	100.0	100.0	100.0
Discordant results (*n*)																								
Major error	1	2	2																1					
Very major error	5	3	3										1	1	1									
Discordant results (%)																								
Major error	1.2	2.4	2.4	0.0	0.0	0.0	0.0	0.0	0.0	0.0	0.0	0.0	0.0	0.0	0.0	0.0	0.0	0.0	1.2	0.0	0.0	0.0	0.0	0.0
Very major error	6.0	3.6	3.6	0.0	0.0	0.0	0.0	0.0	0.0	0.0	0.0	0.0	1.2	1.2	1.2	0.0	0.0	0.0	0.0	0.0	0.0	0.0	0.0	0.0

a BC, blood culture; ATU, area of technical uncertainty. Very major error, RAST = S and reference AST = R; major error, RAST = R and reference AST = S; minor error, RAST = S or R and reference AST = I.

b Categorical agreement for RAST at 4, 6, and 8 h of incubation compared to EUCAST standardized disk diffusion testing.

**A. baumannii**. The overall categorical agreements with spiked BCs were 99.3%, 99.5%, and 99.5% at 4, 6, and 8 h, respectively. The few VMEs were related to amikacin and co-trimoxazole. A high rate of ATU was observed for these two antibiotics at 4 h, but it declined drastically at 6 and 8 h (Table 7). Unfortunately, only two positive BCs were included during the clinical trial. We did not observe any discordant results (Table S1). As documented for E. coli and K. pneumoniae, the results of the spiked BCs predicted the findings from the clinical trial.

**TABLE 7:** *Acinetobacter baumanni* spiked BC results (*n* = 63 isolates)^*a*^

*A. baumannii* spiked BC parameters	Imipenem			Meropenem			Ciprofloxacin^*c*^			Levofloxacin			Gentamicin			Amikacin			Cotrimoxazole		
	4 h	6 h	8 h	4 h	6 h	8 h	4 h	6 h	8 h	4 h	6 h	8 h	4 h	6 h	8 h	4 h	6 h	8 h	4 h	6 h	8 h
Isolates by category (*n*)																					
Resistant	48	50	50	46	50	50	47	49	48	47	48	48	47	49	50	26	33	33		43	44
ATU	1	1	1	5	2		3	2	1	3	3	4	4	2	1	35	12	12	48	4	4
Susceptible	12	12	12	10	11	13	11	12	14	11	12	11	10	12	12	18	18	13	16	15	
Inhibition zone not readable	2	0	0	2	0	0	2	0	0	2	0	0	2	0	0	2	0	0	2	0	0
Isolates by category (%)																					
Resistant	78.7	79.4	79.4	75.4	79.4	79.4	77.0	77.8	76.2	77.0	76.2	76.2	77.0	77.8	79.4	42.6	52.4	52.4	0.0	68.3	69.8
ATU	1.6	1.6	1.6	8.2	3.2	0.0	4.9	3.2	1.6	4.9	4.8	6.3	6.6	3.2	1.6	57.4	19.0	19.0	78.7	6.3	6.3
Susceptible	19.7	19.0	19.0	16.4	17.5	20.6	18.0	19.0	22.2	18.0	19.0	17.5	16.4	19.0	19.0	0.0	28.6	28.6	21.3	25.4	23.8
Inhibition zone not readable	3.2	0.0	0.0	3.2	0.0	0.0	3.2	0.0	0.0	3.2	0.0	0.0	3.2	0.0	0.0	3.2	0.0	0.0	3.2	0.0	0.0
Categorical agreement (%)^*b*^																					
ATU included	100.0	100.0	100.0	100.0	100.0	100.0	100.0	100.0	100.0	100.0	100.0	100.0	100.0	100.0	100.0	98.4	98.4	98.4	98.4	98.4	98.4
ATU not included	100.0	100.0	100.0	100.0	100.0	100.0	100.0	100.0	100.0	100.0	100.0	100.0	100.0	100.0	100.0	96.2	98.0	98.0	92.3	98.3	98.3
Discordant results (*n*)																					
Major error																					
Very major error																1	1	1	1	1	1
Discordant results (%)																					
Major error	0.0	0.0	0.0	0.0	0.0	0.0	0.0	0.0	0.0	0.0	0.0	0.0	0.0	0.0	0.0	0.0	0.0	0.0	0.0	0.0	0.0
Very major error	0.0	0.0	0.0	0.0	0.0	0.0	0.0	0.0	0.0	0.0	0.0	0.0	0.0	0.0	0.0	1.6	1.6	1.6	1.6	1.6	1.6

a BC, blood culture; ATU, area of technical uncertainty. Very major error, RAST = S and reference AST = R; major error, RAST = R and reference AST = S; minor error, RAST = S or R and reference AST = I.

b Categorical agreement for RAST at 4, 6 and 8 h of incubation compared to EUCAST standardized disk diffusion testing.

c Isolates with zone diameters greater than the ATU interval were reported “Susceptible, increased exposure” (I).

**P. aeruginosa**. The overall categorical agreements with spiked BCs were 95.1% and 97.2% at 6 and 8 h, respectively. The number of MEs was high for cefepime at 6 h, thus impacting the categorical agreement for this antibiotic, which was substantively less than 95%. However, this number deceased significantly at 8 h (Table 8). The same observation was made regarding ceftazidime, where a categorical agreement stood at above 95% at 8 h compared to that at 6 h. Importantly, the categorical agreement for imipenem did not improve with incubation time, exhibiting a decline of 3% and still being less than 90%. The VMEs observed for imipenem, ciprofloxacin, and tobramycin could be partially explained by heteroresistant populations. Few colonies were visible within the inhibition halo of standardized disk diffusion testing (Fig. S2). These colonies were not observed by RAST at 8 h because a longer incubation time was required.

**TABLE 8:** *Pseudomonas aeruginosa* spiked BC results (*n* = 129 isolates)^*a*^

*P. aeruginosa* results	Piperacillin-tazobactam^*b*^		Ceftazidime^*b*^		Cefepime^*b*^		Imipenem^*b*^		Meropenem		Ciprofloxacin^*b*^		Levofloxacin		Amikacin		Tobramycin	
	6 h	8 h	6 h	8 h	6 h	8 h	6 h	8 h	6 h	8 h	6 h	8 h	6 h	8 h	6 h	8 h	6 h	8 h
Isolates by category (*n*)																		
Resistant	20	17	13	13	37	13	11	16	10	8	7	10	15	16	13	6	3	2
ATU	43	37	38	23	42	25	5	6	9	9	8	10	8	12	17	15	3	6
Susceptible	62	75	74	93	46	91	109	107	106	112	110	109	102	101	95	108	119	121
Inhibition zone not readable	4	0	4	0	4	0	4	0	4	0	4	0	4	0	4	0	4	0
Isolates by category (%)																		
Resistant	16.0	13.2	10.4	10.1	29.6	10.1	8.8	12.4	8.0	6.2	5.6	7.8	12.0	12.4	10.4	4.7	2.4	1.6
ATU	34.4	28.7	30.4	17.8	33.6	19.4	4.0	4.7	7.2	7.0	6.4	7.8	6.4	9.3	13.6	11.6	2.4	4.7
Susceptible	49.6	58.1	59.2	72.1	36.8	70.5	87.2	82.9	84.8	86.8	88.0	84.5	81.6	78.3	76.0	83.7	95.2	93.8
Inhibition zone not readable	3.1	0.0	3.1	0.0	3.1	0.0	3.1	0.0	3.1	0.0	3.1	0.0	3.1	0.0	3.1	0.0	3.1	0.0
Categorical agreement (%)^*c*^																		
ATU included	96.0	98.4	93.6	96.1	80.8	97.7	92.8	89.9	98.4	99.2	97.6	96.9	98.4	98.4	98.4	99.2	98.4	98.4
ATU not included	93.9	97.8	90.8	95.3	71.1	97.1	92.5	89.4	98.3	99.2	97.4	96.6	98.3	98.3	98.1	99.1	98.4	98.4
Discordant results (*n*)																		
Major error	5	1	6	4	24	2	4	9	2	1	1	2	1	1	2	1		
Very major error		1	2	1		1	5	4			2	2	1	1			2	2
Discordant results (%)																		
Major error	3.9	0.8	4.7	3.1	18.6	1.6	3.1	7.0	1.6	0.8	0.8	1.6	0.8	0.8	1.6	0.8	0.0	0.0
Very major error	0.0	0.8	1.6	0.8	0.0	0.8	3.9	3.1	0.0	0.0	1.6	1.6	0.8	0.8	0.0	0.0	1.6	1.6

a BC, blood culture; ATU, area of technical uncertainty. Very major error, RAST = S and reference AST = R; major error, RAST = R and reference AST = S; minor error, RAST = S or R and reference AST = I.

b Isolates with zone diameters greater than the ATU interval were reported as “Susceptible, increased exposure” (I).

c Categorical agreement for RAST at 4, 6 and 8 h of incubation compared to EUCAST standardized disk diffusion testing.

During the clinical phase, only 12 independent positive BCs (9 patients) were included. We observed one VME for imipenem, ceftazidime, and piperacillin-tazobactam at the two time points (Table S2). Despite the small number of P. aeruginosa-positive BCs received during the clinical phase, the results supported the observation made for the spiked BCs. The overall categorical agreement for all antibiotics tested during the clinical trial was 97.2% at 6 and 8 h.

**S. aureus**. Among the S. aureus included in this study, there were 20 MRSA isolates (20/275; 7.3%). All these isolates were correctly identified as methicillin-resistant after 4 h using a cefoxitin disk. The overall categorical agreements with spiked BCs were 100%, 99.7%, and 99.8% at 4, 6, and 8 h, respectively. A high rate of ATU was observed for norfloxacin at 4 h, but it declined drastically at 6 and 8 h (Table 9). The results of the clinical trial were comparable with those of the spiked BCs. The overall categorical agreements for all the antibiotics tested during the clinical trial were 99.8%, 99.6%, and 99.6% at 4, 6, and 8 h, respectively (Table 10).

**TABLE 9:** *Staphylococcus aureus* spiked BC results (*n* = 148 isolates)^*a*^

*S. aureus* spiked BC parameters	Cefoxitin			Clindamycin			Norfloxacin			Gentamicin		
	4 h	6 h	8 h	4 h	6 h	8 h	4 h	6 h	8 h	4 h	6 h	8 h
Isolates by category (*n*)												
Resistant	11	17	17	0	1	1	0	28	23	7	9	8
ATU	1	0	0	9	7	3	54	7	5	24	19	17
Susceptible	117	131	131	120	140	144	75	113	120	99	120	123
Inhibition zone not readable	19	0	0	19	0	0	19	0	0	18	0	0
Isolates by category (%)												
Resistant	8.5	11.5	11.5	0.0	0.7	0.7	0.0	18.9	15.5	5.4	6.1	5.4
ATU	0.8	0.0	0.0	7.0	4.7	2.0	41.9	4.7	3.4	18.5	12.8	11.5
Susceptible	90.7	88.5	88.5	93.0	94.6	97.3	58.1	76.4	81.1	76.2	81.1	83.1
Inhibition zone not readable	12.8	0.0	0.0	12.8	0.0	0.0	12.8	0.0	0.0	12.2	0.0	0.0
Categorical agreement (%)^*b*^												
ATU included	100.0	100.0	100.0	100.0	100.0	100.0	100.0	99.3	99.3	100.0	99.3	100.0
ATU not included	100.0	100.0	100.0	100.0	100.0	100.0	100.0	99.3	99.3	100.0	99.3	100.0
Discordant results (n)												
Major error											1	
Very major error								1	1			
Discordant results (%)												
Major error	0.0	0.0	0.0	0.0	0.0	0.0	0.0	0.0	0.0	0.0	0.7	0.0
Very major error	0.0	0.0	0.0	0.0	0.0	0.0	0.0	0.7	0.7	0.0	0.0	0.0

a BC, blood culture; ATU, area of technical uncertainty. Very major error, RAST = S and reference AST = R; major error, RAST = R and reference AST = S; minor error, RAST = S or R and reference AST = I.

b Categorical agreement for RAST at 4, 6 and 8 h of incubation compared to EUCAST standardized disk diffusion testing.

**TABLE 10:** *Staphylococcus aureus* clinical trial results (*n* = 127 independent positive BCs from 27 patients)^*a*^

*S. aureus* clinical trial parameters	Cefoxitin			Clindamycin			Norfloxacin			Gentamicin		
	4 h	6 h	8 h	4 h	6 h	8 h	4 h	6 h	8 h	4 h	6 h	8 h
Isolates by category (*n*)												
Resistant	22	22	22		2	1		27	28	20	21	24
ATU	1	0	0	7	7	5	44	6	4	17	12	23
Susceptible	101	105	105	117	118	121	80	94	95	87	94	80
Inhibition zone not readable	3	0	0	3	0	0	3	0	0	3	0	0
Isolates by category (%)												
Resistant	17.7	17.3	17.3		1.6	0.8		21.3	22.0	16.1	16.5	18.9
ATU	0.8	0.0	0.0	5.6	5.5	3.9	35.5	4.7	3.1	13.7	9.4	18.1
Susceptible	81.5	82.7	82.7	94.4	92.9	95.3	64.5	74.0	74.8	70.2	74.0	63.0
Inhibition zone not readable	2.4	0.0	0.0	2.4	0.0	0.0	2.4	0.0	0.0	2.4	0.0	0.0
Categorical agreement (%)^*b*^												
ATU included	100.0	100.0	100.0	99.2	99.2	99.2	100.0	99.2	99.2	100.0	100.0	100.0
ATU not included	100.0	100.0	100.0	99.1	99.2	99.2	100.0	99.2	99.2	100.0	100.0	100.0
Discordant results (*n*)												
Major error				1	1	1						
Very major error								1	1			
Discordant results (%)												
Major error	0.0	0.0	0.0	0.8	0.8	0.8	0.0	0.0	0.0	0.0	0.0	0.0
Very major error	0.0	0.0	0.0	0.0	0.0	0.0	0.0	0.8	0.8	0.0	0.0	0.0

a BC, blood culture; ATU, area of technical uncertainty. Very major error, RAST = S and reference AST = R; major error, RAST = R and reference AST = S; minor error, RAST = S or R and reference AST = I.

b Categorical agreement for RAST at 4, 6 and 8 h of incubation compared to EUCAST standardized disk diffusion testing.

**E. faecalis and E. faecium**. The overall categorical agreement for E. faecalis with spiked BCs was 99.1% at 4, 6, and 8 h (Table 11). The few MEs observed were related to gentamicin. According to EUCAST RAST breakpoints (version 3.0), the rate of ATU for gentamicin was also high and declined only slightly at 6 and 8 h. The overall categorical agreement for E. faecium with spiked BCs was 100.0% at 4, 6, and 8 h (Table 12). The results of the clinical trial reinforced those of the spiked BCs (Tables 13 and 14).

**TABLE 11:** *Enterococcus faecalis* spiked BC results (*n* = 66 isolates)^*a*^

*E. faecalis* spiked BC parameters	Ampicillin			Imipenem^*b*^			Vancomycin			Gentamicin			Linezolid		
	4 h	6 h	8 h	4 h	6 h	8 h	4 h	6 h	8 h	4 h	6 h	8 h	4 h	6 h	8 h
Isolates by category (*n*)															
Resistant							5	6	6	21^*c*^	23^*c*^	21^*c*^			
ATU	2						57	60	60	32	28	28	22	10	11
Susceptible	60	66	66	62	66	66				9	15	17	40	56	55
Inhibition zone not readable	4	0	0	4	0	0	4	0	0	4	0	0	4	0	0
Isolates by category (%)															
Resistant	0.0	0.0	0.0	0.0	0.0	0.0	8.1	9.1	9.1	33.9	34.8	31.8	0.0	0.0	0.0
ATU	3.2	0.0	0.0	0.0	0.0	0.0	91.9	90.9	90.9	51.6	42.4	42.4	35.5	15.2	16.7
Susceptible	96.8	100.0	100.0	100	100	100	0.0	0.0	0.0	14.5	22.7	25.8	64.5	84.8	83.3
Inhibition zone not readable	6.1	0.0	0.0	6.1	0.0	0.0	6.1	0.0	0.0	6.1	0.0	0.0	6.1	0.0	0.0
Categorical agreement (%)^*d*^															
ATU included	100.0	100.0	100.0	100.0	100.0	100.0	100.0	100.0	100.0	95.0	95.0	95.0	100.0	100.0	1 00.0
ATU not included	100.0	100.0	100.0	100.0	100.0	100.0	100.0	100.0	100.0	94.6	95.0	95.0	100.0	100.0	100.0
Discordant results (*n*)															
Major error										3	3	3			
Very major error															
Discordant results (%)															
Major error	0.0	0.0	0.0	0.0	0.0	0.0	0.0	0.0	0.0	4.5	4.5	4.5	0.0	0.0	0.0
Very major error	0.0	0.0	0.0	0.0	0.0	0.0	0.0	0.0	0.0	0.0	0.0	0.0	0.0	0.0	0.0

a BC, blood culture; ATU, area of technical uncertainty. Very major error, RAST = S and reference AST = R; major error, RAST = R and reference AST = S; minor error, RAST = S or R and reference AST = I.

b Isolates with zone diameters greater than the ATU interval were reported as “Susceptible, increased exposure” (I).

c High-level aminoglycoside resistance.

d Categorical agreement for RAST at 4, 6 and 8 h of incubation compared to EUCAST standardized disk diffusion testing.

**TABLE 12:** *Enterococcus faecium* spiked BC results (*n* = 48 isolates)^*a*^

*E. faecium* spiked BC parameters	Ampicillin			Imipenem			Vancomycin			Gentamicin			Linezolid		
	4 h	6 h	8 h	4 h	6 h	8 h	4 h	6 h	8 h	4 h	6 h	8 h	4 h	6 h	8 h
Isolates by category (*n*)															
Resistant	45	45	45	47	46	46	22	23	23	20^*c*^	20^*c*^	20^*c*^			
ATU				1	2	2	26	25	25	6		3		18	9
Susceptible	3	3	3							22	28	25		30	39
Inhibition zone not readable	0	0	0	0	0	0	0	0	0	0	0	0		0	0
Isolates by category (%)															
Resistant	93.8	93.8	93.8	97.9	95.8	95.8	45.8	47.9	47.9	41.7	41.7	41.7		0	0
ATU	0.0	0.0	0.0	2.1	4.2	4.2	54.2	52.1	52.1	12.5	0.0	6.3		37.5	18.8
Susceptible	6.3	6.3	6.3	0.0	0.0	0.0	0.0	0.0	0.0	45.8	58.3	52.1		62.5	81.3
Inhibition zone not readable	0.0	0.0	0.0	0.0	0.0	0.0	0.0	0.0	0.0	0.0	0.0	0.0		0.0	0.0
Categorical agreement (%)^*b*^															
ATU included	100.0	100.0	100.0	100.0	100.0	100.0	100.0	100.0	100.0	100.0	100.0	100.0		100.0	100.0
ATU not included	100.0	100.0	100.0	100.0	100.0	100.0				100.0	100.0	100.0		100.0	100.0
Discordant results															
Major error															
Very major error															

a BC, blood culture; ATU, area of technical uncertainty. Very major error, RAST = S and reference AST= R; major error, RAST = R and reference AST = S; minor error, RAST = S or R and reference AST = I.

b Categorical agreement for RAST at 4, 6 and 8 h of incubation compared to EUCAST standardized disk diffusion testing.

c High-level aminoglycoside resistance.

**TABLE 13:** *Enterococcus faecalis* clinical trial results (*n* = 50 independent positive BCs from 13 patients)^*a*^

*E. faecalis* clinical trial parameters	Ampicillin			Imipenem^*b*^			Vancomycin			Gentamicin			Linezolid		
	4 h	6 h	8 h	4 h	6 h	8 h	4 h	6 h	8 h	4 h	6 h	8 h	4 h	6 h	8 h
Isolates by category (*n*)															
Resistant										25^*c*^	24^*c*^	21^*c*^			
ATU	8	4	3				46	50	50	12	20	19	9	5	7
Susceptible	38	46	47	47	50	50				9	6	10	22	29	27
Inhibition zone not readable	4	0	0	4	0	0	4	0	0	4	0	0	3	0	0
Isolates by category (%)															
Resistant	0.0	0.0	0.0	0.0	0.0	0.0	0.0	0.0	0.0	54.8	61.8	58.8	0.0	0.0	0.0
ATU	17.4	8.0	6.0	0.0	0.0	0.0	100	100	100	22.6	35.3	26.5	29.0	14.7	20.6
Susceptible	82.6	92.0	94.0	100.0	100.0	100.0	0.0	0.0	0.0	22.6	2.9	14.7	71.0	85.3	79.4
Inhibition zone not readable	8.0	0.0	0.0	8.0	0.0	0.0	8.0	0.0	0.0	8.0	0.0	0.0	8.8	0.0	0.0
Categorical agreement (%)^*d*^															
ATU included	100.0	100.0	100.0	100.0	100.0	100.0				95.7	96.0	96.0	100.0	100.0	100.0
ATU not included	100.0	100.0	100.0	100.0	100.0	100.0				94.1	93.3	93.5	100.0	100.0	100.0
Discordant results (*n*)															
Major error										1	1	1			
Very major error										1	1	1			
Discordant results (%)															
Major error	0.0	0.0	0.0	0.0	0.0	0.0	0.0	0.0	0.0	2.0	2.0	2.0	0.0	0.0	0.0
Very major error	0.0	0.0	0.0	0.0	0.0	0.0	0.0	0.0	0.0	2.0	2.0	2.0	0.0	0.0	0.0

a BC, blood culture; ATU, area of technical uncertainty. Very major error, RAST = S and reference AST = R; major error, RAST = R and reference AST = S; minor error, RAST = S or R and reference AST = I.

b Isolates with zone diameters greater than the ATU interval were reported as “Susceptible, increased exposure” (I).

c High-level aminoglycoside resistance.

d Categorical agreement for RAST at 4, 6 and 8 h of incubation compared to EUCAST standardized disk diffusion testing.

**TABLE 14:** *Enterococcus faecium* clinical trial results (*n* = 44 independent positive BCs from 13 patients)^*a*^

*E. faecium* clinical trial parameters	Ampicillin			Imipenem			Vancomycin			Gentamicin			Linezolid		
	4 h	6 h	8 h	4 h	6 h	8 h	4 h	6 h	8 h	4 h	6 h	8 h	4 h	6 h	8 h
Isolates by category (*n*)															
Resistant	31	25	29	38	36	36	1	1	1	13^*c*^	13^*c*^	13^*c*^			
ATU	1	7	3	6	8	8	43	43	43	11	12	12		25	11
Susceptible	12	12	12	0	0	0	0	0	0	20	19	19		19	33
Inhibition zone not readable	0	0	0	0	0	0	0	0	0	0	0	0		0	0
Isolates by category (%)															
Resistant	70.5	56.8	65.9	86.4	81.8	81.8	2.3	2.3	2.3	29.5	29.5	29.5		0.0	0.0
ATU	2.3	15.9	6.8	13.6	18.2	18.2	97.7	97.7	97.7	25.0	27.3	27.3		56.8	25.0
Susceptible	27.3	27.3	27.3	0.0	0.0	0.0	0.0	0.0	0.0	45.5	43.2	43.2		43.2	75.0
Inhibition zone not readable	0.0	0.0	0.0	0.0	0.0	0.0	0.0	0.0	0.0	0.0	0.0	0.0		0.0	0.0
Categorical agreement (%)^*b*^															
ATU included	100.0	100.0	100.0	100.0	100.0	100.0	100.0	100.0	100.0	100.0	97.7	95.5		100.0	100.0
ATU not included	100.0	100.0	100.0	100.0	100.0	100.0	100.0	100.0	100.0	100.0	96.9	93.8		100.0	100.0
Discordant results (*n*)															
Major error											1	2			
Very major error															
Discordant results (%)															
Major error	0.0	0.0	0.0	0.0	0.0	0.0	0.0	0.0	0.0	0.0	2.3	4.5	0.0	0.0	0.0
Very major error	0.0	0.0	0.0	0.0	0.0	0.0	0.0	0.0	0.0	0.0	0.0	0.0	0.0	0.0	0.0

a BC, blood culture; ATU, area of technical uncertainty. Very major error, RAST = S and reference AST = R; major error, RAST = R and reference AST = S; minor error, RAST = S or R and reference AST = I.

b Categorical agreement for RAST at 4, 6 and 8 h of incubation compared to EUCAST standardized disk diffusion testing.

c High-level aminoglycoside resistance.

Among the 30 non-duplicate VRE isolates analyzed in this study, only one (VanB phenotype) had a vancomycin-inhibition zone diameter in the ATU at 4 h (12 mm). However, it was categorized as vancomycin-resistant (<12 mm) at both 6 and 8 h. The vancomycin-inhibition zone diameters ranged from 6 to 11 mm at 4 h for the other 29 VRE isolates included in this study. Accordingly, they were all categorized as vancomycin-resistant. No discordant results were observed for vancomycin at the different time points.

For the isolates with the VanA phenotype, vancomycin MICs ranged between 32 and 256 mg/L. For the isolates with the VanB phenotype, vancomycin MICs ranged between 6 and 256 mg/L.

### Quality control.

The RAST procedure using reference ATCC strains was repeated several times throughout the experiments. QC values for the RAST method were always within the EUCAST RAST published ranges (Fig. S3).

### Screening and confirmation of ESBL by RAST.

Screening for ESBL producers using the RAST method was performed according to EUCAST RAST screening cutoff values at 4, 6, and 8 h for cefotaxime and ceftazidime. All K. pneumoniae isolates determined to be resistant to cefotaxime and/or ceftazidime by EUCAST standardized disk diffusion testing were categorized as resistant or in the ATU by RAST.

For E. coli, 3 VMEs were observed for ceftazidime at the different time points. However, all the cefotaxime-resistant isolates were categorized as resistant or in the ATU by RAST. For the 122 ESBL-producer isolates showing a positive DDST20 using standardized disk diffusion testing, the performance of the DDST20 at 4, 6, and 8 h using the RAST method was as follows: 67% positivity at 4 h, reaching 100% at 6 and 8 h (Fig. S4).

### Screening for carbapenemase production by RAST.

All E. coli and K. pneumoniae isolates determined to be resistant to meropenem by EUCAST standardized disk diffusion testing were categorized as resistant or in the ATU by RAST. Few MEs were observed for meropenem at the different time points in both species without any impact on the sensitivity of CPE screening. Considering the low rate of meropenem-ATU (<5%), isolates expressing this profile were also systematically screened for carbapenemase production. Hence, screen-positive and meropenem-ATU isolates were subjected to molecular confirmatory assays.

### Inducible clindamycin resistance.

When the clindamycin 2-μg disk and erythromycin 15-μg disk were placed 9 mm apart (edge to edge), the percentages of positive Dtest among the 49 non-duplicate erythromycin-resistant S. aureus included in this study were 8.2% (4/49), 75.5% (37/49), and 83.7% (41/49) at 4, 6, and 8 h, respectively. When the clindamycin 2-μg disk and erythromycin 15-μg disk were placed 12 mm apart (edge to edge) the percentages of positive Dtest were 12.2% (6/49), 85.7% (42/49), and 100% (49/49) at 4, 6, and 8 h, respectively (Fig. S5).

## DISCUSSION

Despite the development of new and promising methods for antimicrobial susceptibility testing based on different technologies, standard bacterial growth-based methods relying on disc diffusion or broth dilution remain widely used in clinical microbiology laboratories. The slowness of these methods and their traditional requirement of pure cultures are largely balanced by their flexibility, cost-effectiveness, and accuracy for detecting known and even new resistance mechanisms (11). However, shorter times for delivering accurate antimicrobial susceptibility results remain one of the major objectives to be achieved. This has become urgent with the steady increase in antimicrobial resistance rates. In several instances, empirical therapy is not tailored to the microorganism causing the infection (10). Thereby, improving the duration until effective and personalized therapy is administered in patients remains a challenge. During the last few years, commercially available and in-house methods have been developed for RAST directly from positive blood cultures. The main purpose of this study was to determine the diagnostic accuracy and implementation constraints of fully automated EUCAST RAST using the Copan system. We paid special attention to including a large number of clinical isolates expressing diverse resistant phenotypes during the spiked BC phase. Our first observation was that the results of the spiked BCs precisely predicted the results of the clinical trial. For E. coli, the categorical agreement ranged between 97.4% and 100% for all the antibiotics tested, except for amikacin at 4 h, which explains the MEs observed. According to spiked BC and clinical trial results, except for amikacin, all of the antibiotics tested could be released at 4 h with high confidence. Regarding amikacin, the results could be released only at 6 h. Moreover, the high rate of ATU observed for piperacillin-tazobactam and amikacin will impair their usage at 4 h for many cases. For K. pneumoniae, the entire drug panel tested can be released at 4 h, even for MDR isolates. Unfortunately, only 2 A. baumannii isolates were included in the clinical trial, but the results of the spiked BCs confirm that the entire tested drug panel can be safely released at 4 h. The high rate of ATU for co-trimoxazole at 4 h significantly declined from 78.7% to 6.3% at 6 h. Unlike the other species, P. aeruginosa posed several challenges. The categorical agreement for imipenem was less than 95% at 6 h and did not improve by 8 h. According to this study, using the RAST for imipenem is not accurate and this antibiotic should be removed from the panel of antibiotics to be tested for P. aeruginosa. Additionally, piperacillin-tazobactam, ceftazidime, and cefepime cannot be released at 6 h, so administration should be held off until the next time point because the categorical agreement is substantially improved at 8 h. Therefore, greater attention should be paid in interpreting the P. aeruginosa panel when it is tested by RAST. For S. aureus, cefoxitin enables the identification of MRSA with very high accuracy at 4 h, when the inhibition zone is readable. A high rate of ATU was observed at 4 h for norfloxacin, but it had declined from 74.9% to 4.7% by 6 h. The entire drug panel can therefore be released at 4 h. For E. faecalis and E. faecium, the categorical agreement was >95% for all antibiotics tested except for gentamicin. All the 30 VRE isolates analyzed in this study were categorized as vancomycin-resistant at 6 h. However, according to EUCAST recommendations, the vancomycin disk does not correctly predict resistance in *vanB*-positive isolates with low MICs, which explains the high rate of ATU observed using the RAST EUCAST Breakpoint Tables version 3.0. Therefore, special attention should be paid to isolates within the vancomycin-ATU.

The decision to include all positive BCs from the same patients enabled us to highlight the high repeatability and reproducibility of the RAST. No discordant results at the categorical level were observed among BCs from the same patient.

The fully automated RAST allows inoculated media to be transferred to the incubator immediately after deposition of the antibiotic disks. The medium plates are incubated under optimal growth conditions, served by a stable temperature and adequate atmosphere. The digital images taken at predefined time points can be inspected using magnification. All these elements explain the improved percentages of readable inhibition zones at the different time points. Another positive impact was the lower percentage of ATU compared to that in data published previously using the manual process (12, 15).

The manual processing of the EUCAST RAST and especially the need to read the inhibition zone diameters at strictly defined time points translate into a very labor-intensive method. A previous report highlighted these challenges (12). In contrast, total laboratory automation (TLA) perfectly addresses these constraints by automatically acquiring images at predefined time points. Moreover, the expert system facilitates the integration of different rules, in line with the observations made in this study, to optimize and improve the results released to physicians. For instance, it automatically defines which antibiotic results can be released at 4 h and which can be released at later, well-validated incubation times. The advantages of full automation facilitate the implementation of the RAST directly from positive BCs in routine. In our laboratory, positive BCs are processed by the WASPLab. To further improve the TAT of RAST, we defined a panel of antibiotics covering all species analyzed in this study. Thereby, RAST can be performed without waiting for microscopy results and Gram staining; non-pertinent antibiotics can then be excluded from reporting during the reading phase.

The system used in our laboratory for the fully automated RAST AST is composed of one WASP and a single “air atmosphere” incubator. These incubation constraints (i.e., the CO_2_ requirement) prevented us from using RAST to assess Streptococcus pneumoniae in this study.

### Conclusions.

In our previous report, we validated the full automation of standardized antimicrobial disk diffusion susceptibility testing (11). Nowadays, 97% of our AST panels are performed using this system in routine (17). Based on this positive experience, we extended the use of the fully automated solution to the EUCAST rapid AST method directly from positive blood cultures. The spiked BCs and the clinical study established that the performance of this automation was consistently robust, even for the detection of ESBL, carbapenemase-producing bacteria, and MRSA. In addition, the automation enhances the percentage of readable inhibition zones and reduces the percentage of isolates categorized in the ATU. The fully automated RAST, coupled to the expert system, will substantially improve workflow by reducing hands-on times and the constraints linked to manual workup.

### Ethical approval.

In accordance with local ethical committees, routine clinical laboratories of our institution may use biological sample leftovers for method development after irreversible anonymization of data. The official name of the ethics committee is the “Commission cantonale d’éthique de la recherche” (CCER; https://www.hug-ge.ch/ethique).
