# Supplementary material for: Fully Automated EUCAST Rapid Antimicrobial Susceptibility Testing (RAST) from Positive Blood Cultures: Diagnostic Accuracy and Implementation
Source: J Clin Microbiol. 2022 Sep 29;60(10):e00898-22. doi: 10.1128/jcm.00898-22 (PMC9580353; doi:10.1128/jcm.00898-22)
Supplement: Supplemental file 1 — Supplemental material. Download jcm.00898-22-s0001.pdf, PDF file, 1.6 MB [file jcm.00898-22-s0001.pdf]

Fully-automated EUCAST rapid antimicrobial susceptibility testing (RAST) from positive blood cultures: diagnostic accuracy and implementation

Abdessalam Cherkaoui[1,2*], Didier Schorderet[1], Nouria Azam[1], Luigi Crudeli[1], José Fernandez[1], Gesuele Renzi[1], Adrien Fischer[1], and Jacques Schrenzel[1,3]

[1]Bacteriology Laboratory, Division of Laboratory Medicine, Department of Diagnostics, Geneva University Hospitals, 4 rue Gabrielle-Perret-Gentil, 1205 Geneva, Switzerland

[2]Faculty of Medicine, Geneva, Switzerland

[3]Genomic Research Laboratory, Division of Infectious Diseases, Department of Medicine, Geneva University Hospitals and Faculty of Medicine, Geneva, Switzerland

*__Corresponding author__: Abdessalam CHERKAOUI, PD - PhD - FAMH

Bacteriology Laboratory, Division of Laboratory Medicine, Department of Diagnostics, Geneva University Hospitals, 4 rue Gabrielle-Perret-Gentil, 1205 Geneva, Switzerland

E-mail address: abdessalam.cherkaoui@hcuge.ch

Figure-S1: **Study design**

Spiked NEGATIVE blood culture (BC) bottles

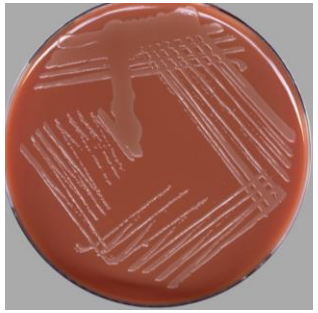

1 mL of a 100-200 CFU/mL solution

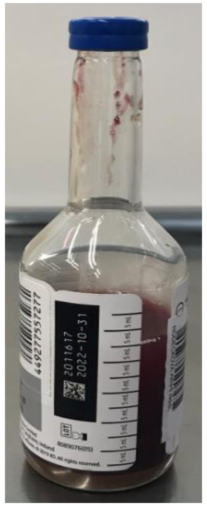

Spiking previously negative blood culture bottles

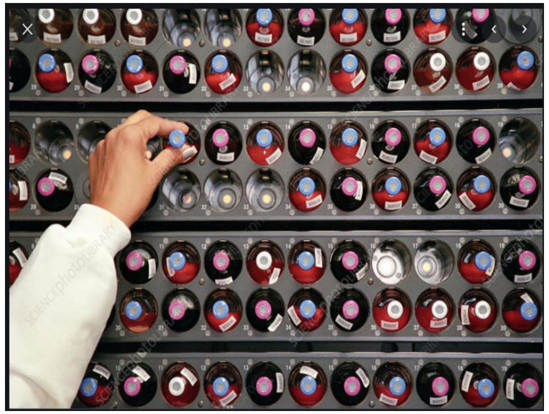

Incubated in the BD BACTEC™ FX

Viable cell counted on Columbia agar for 10 **non-consecutive** positive BCs

Inoculums ranged between 1.3 x 10^8 and 5.6 x 10^8 CFU/ml

Figure-S1: Study design

Spiked NEGATIVE BC bottles / Performing RAST on WASPLab

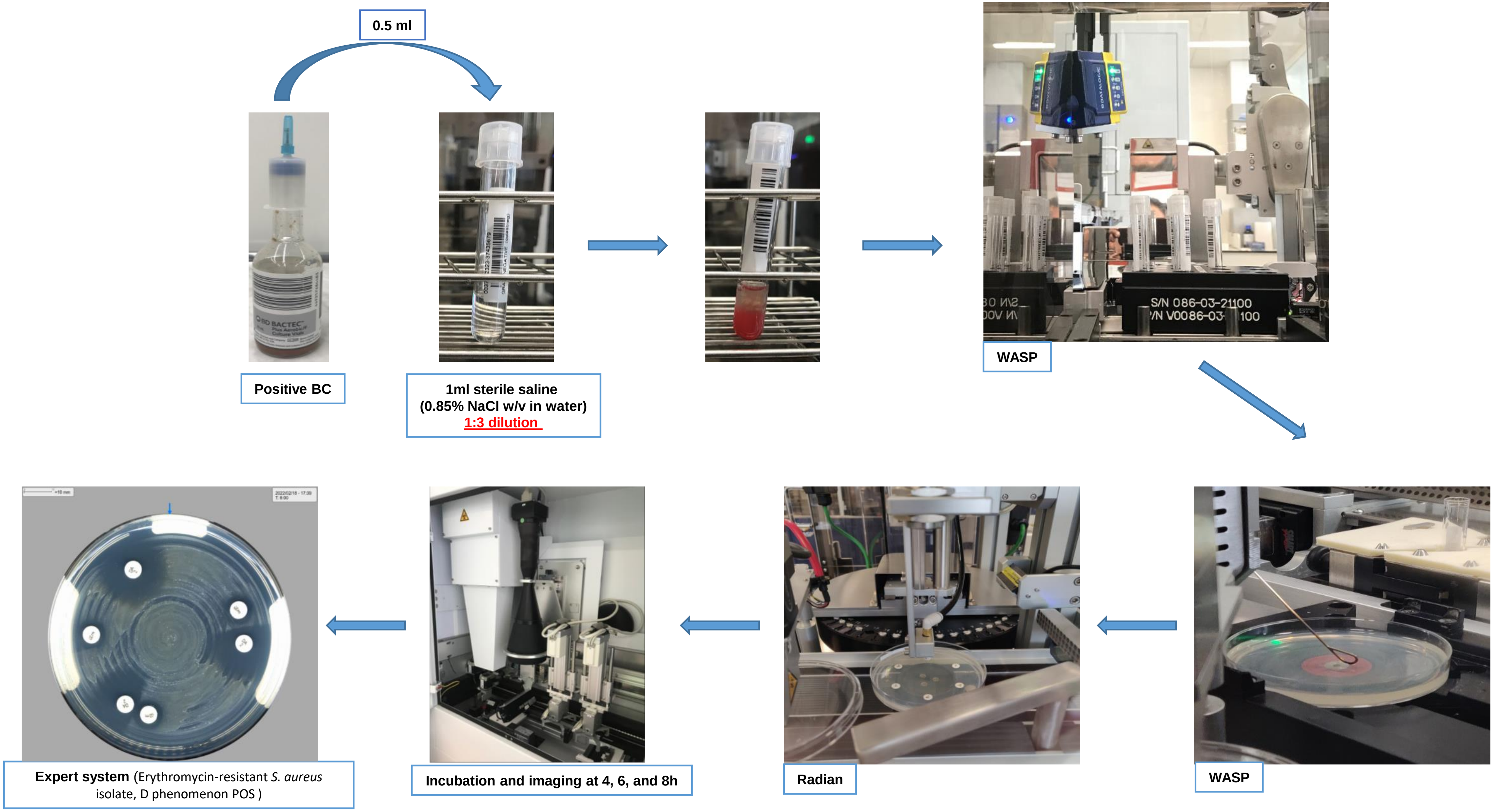

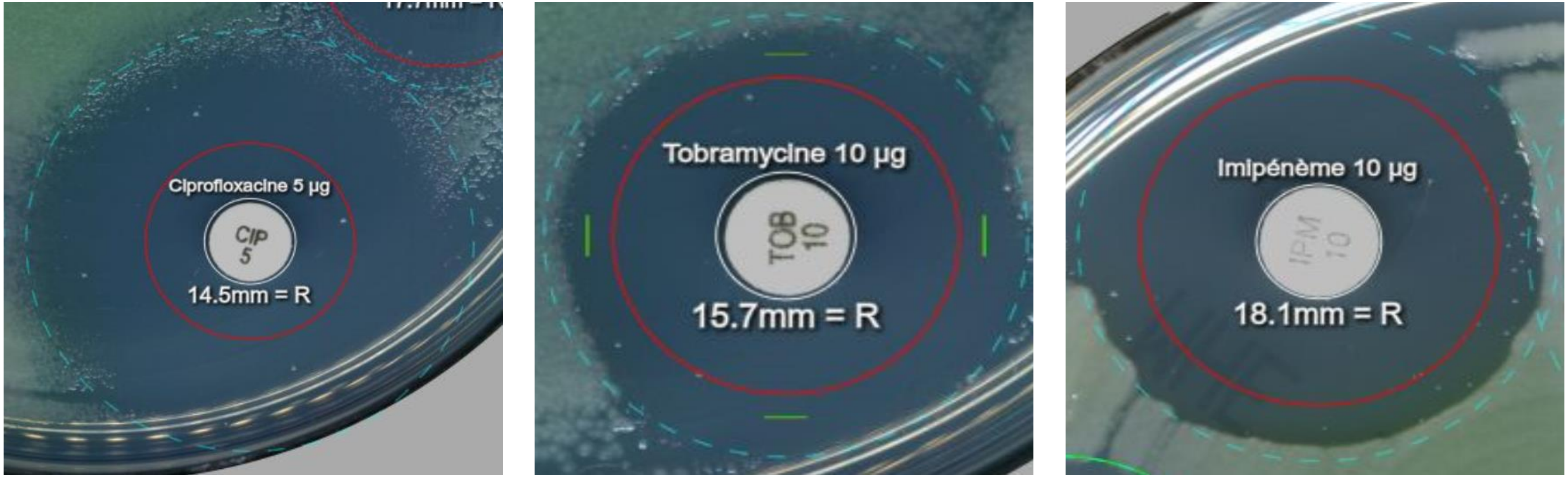

EUCAST standardized disk diffusion testing

Figure-S2:
Heteroresistant populations (colonies visible within the inhibition halo at 16h)
These colonies were not observed using RAST

Figure-S3: Quality control (QC)

The RAST procedure using reference strains was repeated several times throughout the experiments

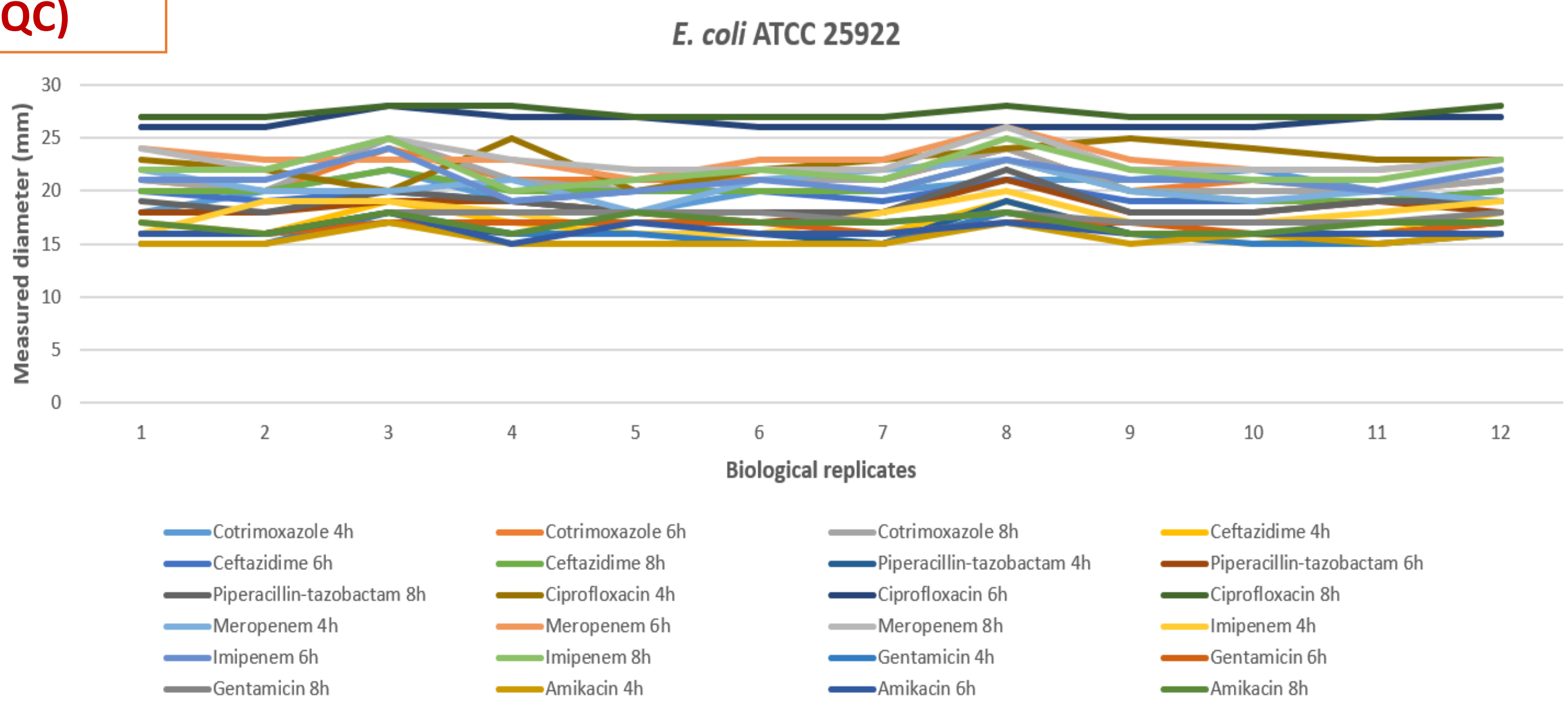

The QC values for the RAST method were within published ranges

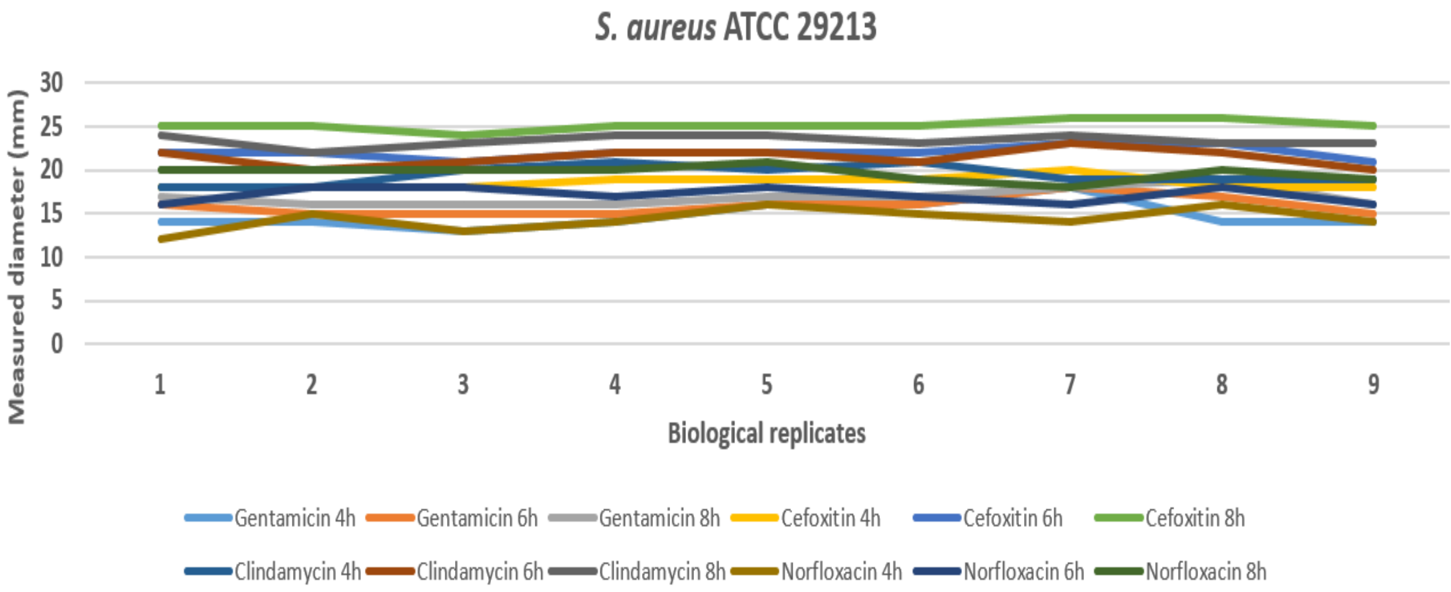

Figure-S4 **Detection of resistance mechanisms and specific resistance by RAST**

ESBL-producing *E. coli* and *K. pneumoniae*

DDS$_{20mm}$ Test performances for the **122 ESBL** isolates analyzed

Results : **67% POS at 4h and 100% POS at 6h**

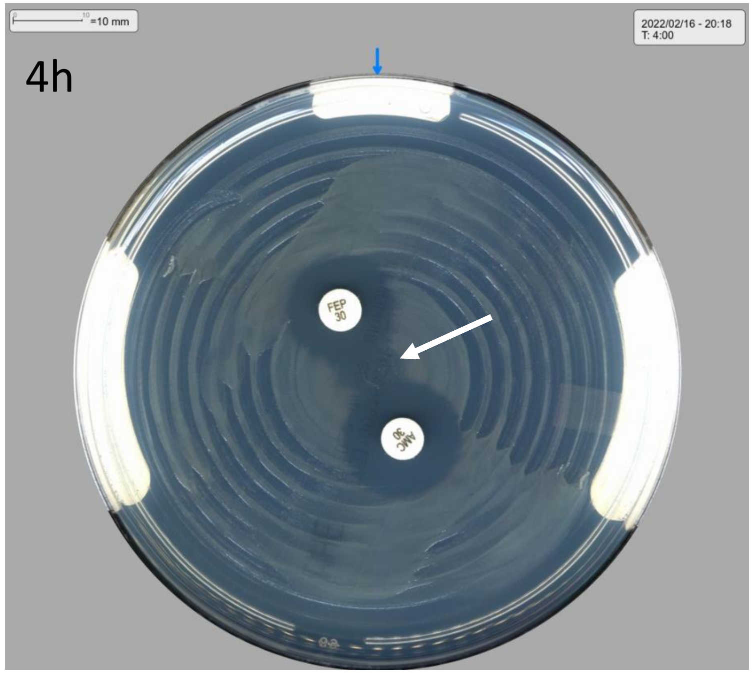

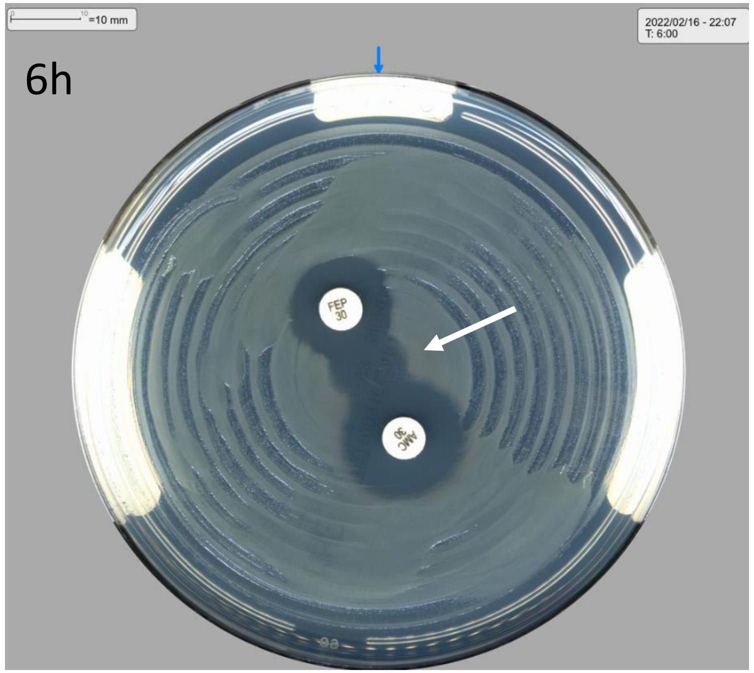

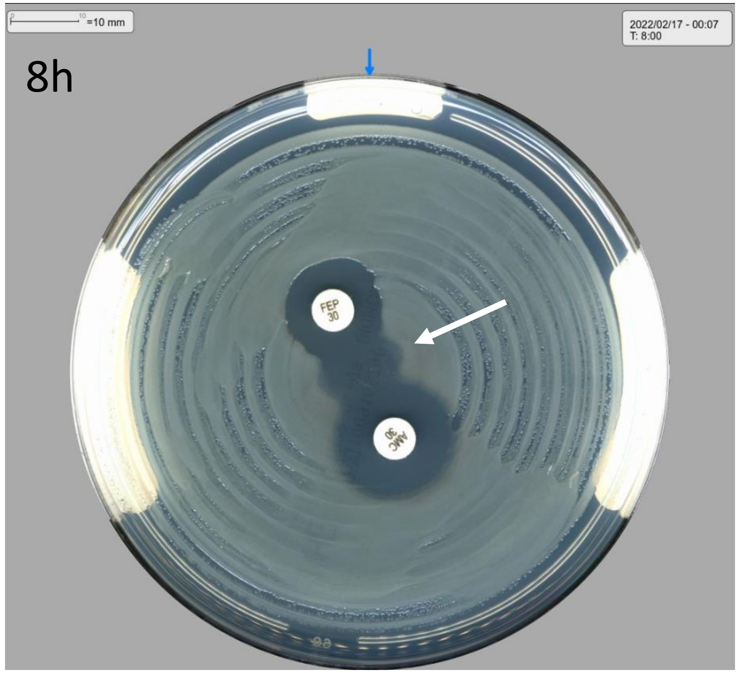

The inhibition zone around FEP disks is enhanced, highly suggesting the production of ESBL (white arrow)

Figure-S5: Inducible clindamycin resistance tests

Number of strains analyzed: **49** non duplicate Erythromycin-Resistant *S. aureus* isolates including **10** MRSA (**Dtest Positive at 16h**)

TEST-1: Clindamycin 2 µg disk and erythromycin 15 µg disk placed **at 9 mm apart (edge to edge)**

	4h	6h	8h
Number of positive tests	4	37	41
Percentage (%)	**8.2**	**75.5**	**83.7**

TEST2: Clindamycin 2 µg disk and erythromycin 15 µg disk placed **at 12mm apart (edge to edge)**

	4h	6h	8h
Number of positive tests	6	42	49
Percentage (%)	**12.2**	**85.7**	**100.0**

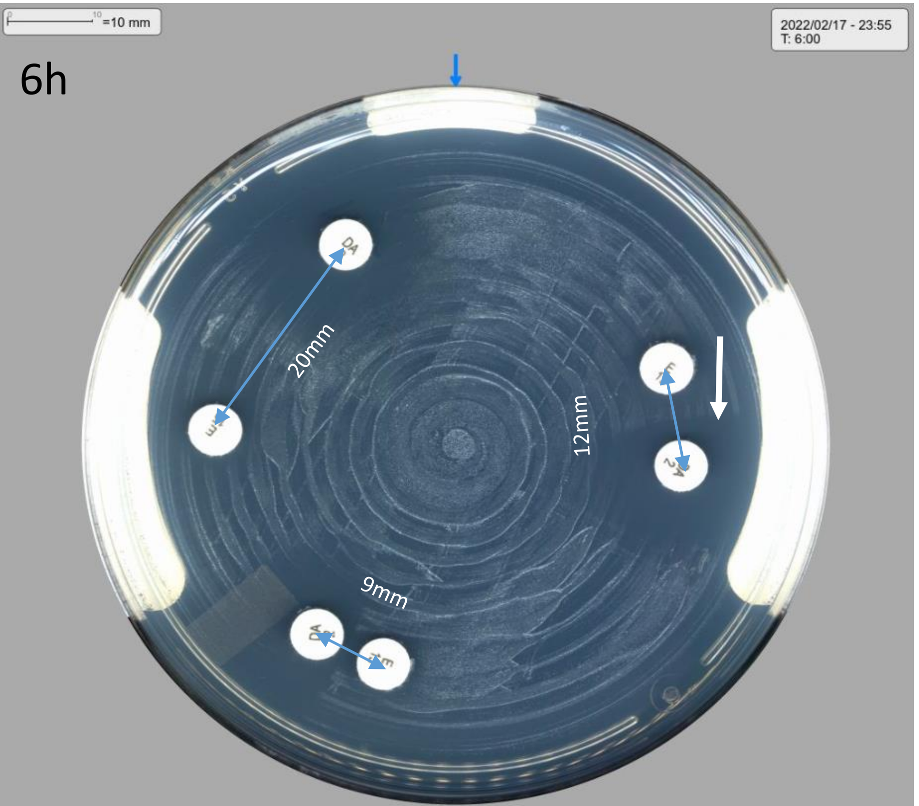

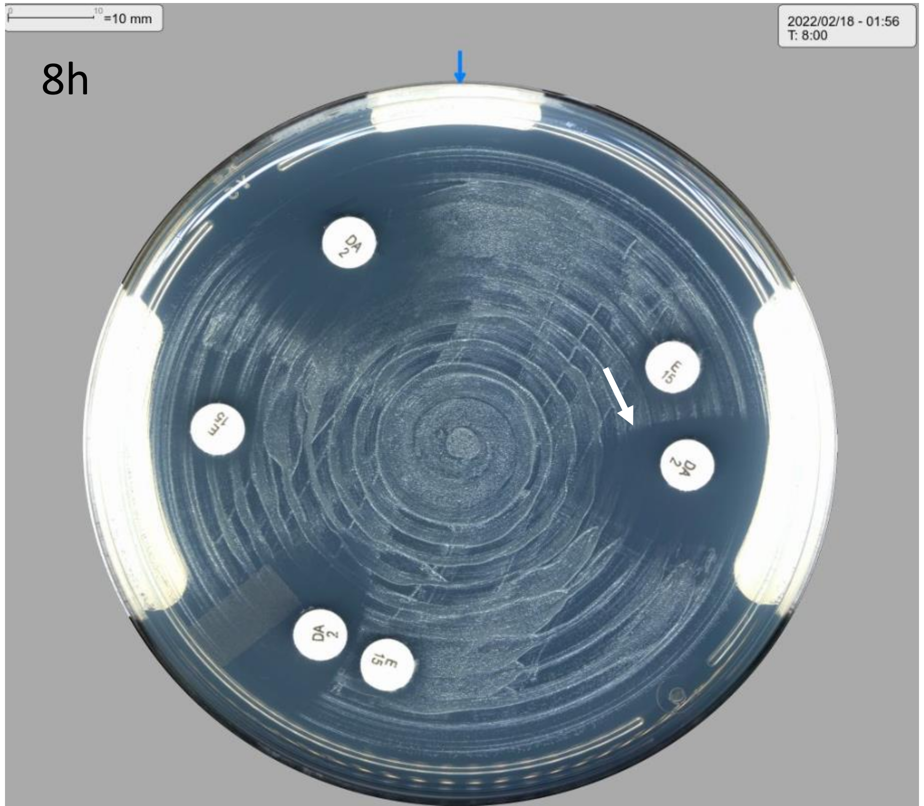

BCs ID	IMP			LEVO			CIPRO			GENT			AMIK			MERO			Tobra			SXT		
	4	6	8	4	6	8	4	6	8	4	6	8	4	6	8	4	6	8	4	6	8	4	6	8
38086140	23	24	25	18	19	19	19	19	20	15	15	16	15	16	16	19	20	21	15	16	16	16	17	17
38086139	24	24	25	18	19	20	19	19	20	15	16	16	16	16	17	19	20	20	15	15	15	16	16	16

Table-S1: The RAST analysis results (inhibition zone diameters) of the 2 BCs positive with *A. baumannii* (Clinical trial)

We did not observe any discordant results

BCs ID	CEF	CEF		CFTA	CFTA		Piptaz	Piptaz		Cipro	Cipro		Levo	Levo		Mero	Mero		IMIP	IMIP		Tobra	Tobra		Amika	Amika	
		6	8		6	8		6	8		6	8		6	8		6	8		6	8		6	8		6	8
36940243	S	18	21	S	14	16	S	15	19	S	24	28	S	21	21	S	17	20	R	20	21	S	20	20	S	17	20
36982079	S	15	20	S	13	15	S	13	16	S	25	27	S	18	20	S	21	23	S	22	21	S	16	18	S	16	17
37274416	R	12	12	R	8	7	R	6	6	R	17	21	R	13	14	R	15	15	R	16	16	S	20	20	S	17	20
37384263	S	19	20	S	15	16	S	13	15	S	24	27	S	20	22	S	25	26	S	21	21	S	16	18	S	18	18
37608546	S	18	21	R	14	15	R	14	16	S	22	27	S	17	19	S	20	23	S	22	21	S	19	20	S	16	18
37608820	S	17	20	R	12	13	R	15	16	S	25	26	S	19	20	S	20	24	S	21	22	S	18	19	S	16	19
37608545	S	17	21	R	14	15	R	18	17	S	23	26	S	20	19	S	22	24	S	22	21	S	19	20	S	18	19
37608819	S	15	20	R	17	18	R	14	15	S	23	26	S	18	20	S	20	23	S	21	21	S	15	20	S	16	17
37614423	R	15	16	S	17	17	S	17	18	R	17	18	R	11	11	S	21	27	S	22	22	S	15	16	S	15	16
37951516	S	18	23	S	15	17	S	18	20	S	22	29	S	21	25	S	19	28	S	22	22	S	17	20	S	18	20
37939217	S	17	22	S	14	18	S	17	19	S	25	27	S	19	21	S	20	24	S	19	20	S	18	19	S	18	19
38025801	S	20	22	S	15	18	S	18	19	S	24	29	S	20	23	S	21	24	S	22	23	S	20	20	S	17	20

- (green) Reference method results
- (grey) RAST results (inhibition zone diameters)
- (yellow) VME

Table-S2: The analysis results of the 12 BCs positive with *P. aeruginosa* (Clinical trial)
